# Long-Term Stimulation of the Left Dorsal Branch of the Thoracic Nerve Improves Ventricular Electrical Remodeling in a Canine Model of Chronic Myocardial Infarction

**DOI:** 10.1007/s10557-024-07602-z

**Published:** 2024-07-09

**Authors:** Juan Hua, Ziyi Xiong, Qiling Kong, Dandan Wang, Jinwei Liu, Huawei Chen, Yuerong Wang, Yan Wu, Qi Chen, Liang Xiong

**Affiliations:** 1 Department of Cardiology, The Second Affiliated Hospital of Nanchang University, 1 Minde Road, Nanchang, Jiangxi 330006 China; 2 College of Animal Science and Technology, Jiangxi Agricultural University, Nanchang, Jiangxi 330045 China

**Keywords:** Autonomic nervous modulation, LDTN stimulation, Electrophysiological remodeling, LSG activity, Neuronal apoptosis

## Abstract

**Purpose:**

To evaluate the ventricular electrophysiologic effects of long-term stimulation of the left dorsal branch of thoracic nerve (LDTN) derived from the left stellate ganglion (LSG) in a canine model of chronic myocardial infarction (MI).

**Methods:**

Seventeen adult male beagles were randomly divided into three groups: the sham group (sham operated, *n* = 6), the MI group (*n* = 6), and the MI + LDTN group (MI plus LDTN stimulation, *n* = 5). The canine model of chronic MI was induced by the occlusion of the left anterior descending artery (LADO). The LDTN was separated and intermittently stimulated immediately after LADO for 2 months. The heart rate variability (HRV) analysis, in vivo electrophysiology, the evaluation of LSG function and neural activity, histological staining, and western blotting (WB) assay were performed to evaluate the effect of LDTN stimulation on the heart.

**Results:**

The canine MI model was successfully established by LADO, and the LDTN was separated and stimulated immediately after LADO. The HRV analysis showed that LDTN stimulation reversed the increased LF value and LF/HF ratio of the MI group. LDTN stimulation prolonged the shortening ERP and APD90, decreased the dispersion of ERP and APD90, and increased the VFT. Additionally, LDTN stimulation inhibits the LSG function and neural activity. Furthermore, LDTN stimulation suppressed the activation of Wnt/β-catenin signaling, which contributed to the LSG neuronal apoptosis by upregulation of pro-apoptotic Bax and downregulation of anti-apoptotic Bcl-2.

**Conclusion:**

LDTN stimulation could attenuate cardiac sympathetic remodeling and improve ventricular electrical remodeling, which may be mediated by suppressing the activated Wnt/β-catenin signaling pathway and then promoting the LSG neuronal apoptosis.

## Introduction

Malignant ventricular arrhythmias (VAs) remain the major and direct cause of sudden cardiac death (SCD) in patients with myocardial infarction (MI) [1]. The occurrences of VAs and SCD are closely associated with cardiac sympathetic hyperactivity [2, 3]. The left stellate ganglion (LSG) is the main target of external cardiac sympathetic innervation of the heart [4). Previous studies have shown that the inhibitory activity of LSG mediated by direct or indirect modulation protects the heart from VAs after MI [5, 6]. The left dorsal branch of the thoracic nerve (LDTN) refers to the extension of the posterior branch of the left thoracic nerve, which is connected to the LSG and contains the postganglionic sympathetic nerve components originating from the LSG. A recent study has shown that long-term high-frequency electrical stimulation of the LDTN causes damage to the LSG neurons and therefore attenuates their activity, thereby decreasing the paroxysmal atrial tachyarrhythmias (PAT) episodes in a canine model of PAT [7]. However, whether LDTN stimulation could reduce the risk of ventricular arrhythmias has not previously been reported and the exact molecular mechanism of LDTN stimulation damaging the LSG neurons remains unknown.

It is well-recognized that apoptosis can be triggered by extrinsic and intrinsic events. Rapid and prolonged excitation of neurons leads to excitotoxic changes and then results in neuronal cell death [8]. The Bcl-2 protein family is regarded as the principal regulators of apoptosis, including the anti-apoptotic protein Bcl-2 and pro-apoptotic protein Bax. Additionally, the Wnt/β-catenin signaling pathway is widely involved in multiple biological processes, including cell proliferation, differentiation, migration, and death [9]. Importantly, the Wnt/β-catenin pathway participates in regulation of neuron apoptosis [10]. Meanwhile, β-catenin is a key regulator of cell survival and death by regulating its downstream targeted genes such as Bcl-2 and Bax [11]. Therefore, we aimed to identify whether long-term LDTN stimulation can induce LSG neuronal cell death through the Wnt/β-catenin pathway, thereby inhibiting cardiac sympathetic tone and ultimately inhibiting the occurrence of VAs and SCD after MI.

## Materials and Methods

### Animal Model and Infarct Induction

**Fig. 1:**
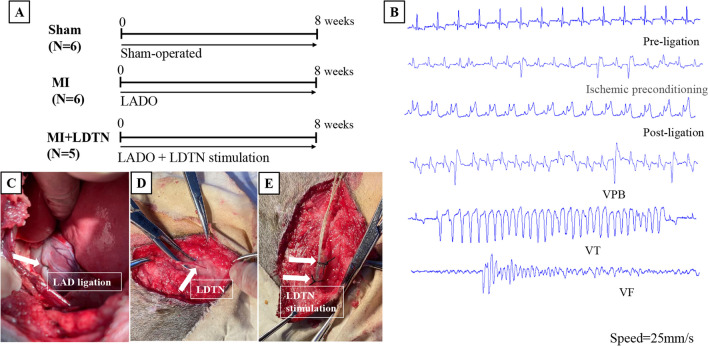
Establishment and evaluation of MI model. **(A)** The protocol of the study. **(B)** Representative images of the dynamic ST-segment elevation and VAs episodes during and after LADO. **(C)** Representative anatomical location of the LADO; **(D)** and **(E)** The anatomy of LDTN and the stimulating electrodes wrapped around the LDTN. MI, myocardial infarction; LADO, occlusion of the left anterior descending branch; LDTN, left dorsal branch of the thoracic nerve; VAs, ventricular arrhythmias; VPB, ventricular premature beat; VT, ventricular tachycardia; VF, ventricular fibrillation

All animal experiments were performed following the Guide for the Care and Use of Laboratory Animals and approved by the Ethics Committee of Jiangxi Agricultural University (ID number: JXAULL-2023–06-08). Seventeen adult male beagles were randomly divided into three groups: the sham group (sham-operated, *n* = 6), the MI group (*n* = 6), and the MI + LDTN group (MI plus LDTN stimulation, *n* = 5) (Fig. 1A). The canine model of chronic MI was induced by occlusion of the left anterior descending artery (LADO) as previously published [12]. Briefly, beagles were sedated with propofol (6–7 mg/Kg, intravenous), and analgesia maintained by inhaled isoflurane (0.8–1.5%). The LAD was ligated with thoracotomy under sterile condition. ST-segment elevation in ECG lead II was used to evaluate the successful construction of MI model.

### LDTN Stimulation

The LDTN at the third intercostal space was separated and stimulated immediately after LADO. The neurostimulator was programmed 14-s ON (10 Hz, 500 μs pulse duration) and 66-s OFF for LDTN stimulation based on the previous study [7]. The output current of the neurostimulator was increased gradually from 1.0 mA to 3.5 mA in 2 weeks, then maintained for 2 months. During the whole process of LDTN stimulation, we have also checked whether the electrode of the neurostimulator was displaced and the animals had any discomfort.

### HRV Analysis

To investigate the effect of LDTN stimulation on autonomic nervous activity, a 5-min ECG with a stable signal was recorded for HRV analysis under sedation and obtained at a similar time of day, including the Poincaré plot maps and spectrum analysis (LF, HF, and LF/HF) at 2 months after MI. The LF value indicates the sympathetic tone, and the HF value indicates the parasympathetic tone, while the LF/HF ratio indicates the relative balance between sympathetic and parasympathetic nerves.

### Evaluation of LSG Function and Neural Activity

Two tungsten-coated microelectrodes were placed into the body of LSG tissue and a ground lead was placed into the chest wall to record LSG neural activity. The neural activity of LSG was measured by the KLB-1 wireless monitoring system (Shanghai, China) and analyzed by the Labchart 8 software.

### In Vivo Electrophysiology Study

Programmed stimulation was performed to measure the effective refractory period (ERP) and action potential duration at 90% repolarization (APD90) of infarct border zone (IBZ), remote area (RB), LV base (LVB), right ventricular apex (RVA), RV base (RVB), and the median RV (RVM). The ventricular fibrillation threshold (VFT) was measured by Burst pacing (10 Hz, 0.1 ms duration, 30 s). The VFT was defined as the lowest voltage inducing the VF which lasts more than 30 s. The ERP was measured by the programmed stimulation of S1S2. The MAP was measured by the programmed stimulation of S1S1 at PCL of 300 ms. The ERP, APD90, and VFT was measured using the KLB-1 wireless monitoring system (Shanghai, China) and analyzed using the Labchart 8 software.

### Histological Staining, Immunofluorescence, and Immunohistochemistry

The LSG of all beagles were harvested and fixed in 10% formaldehyde for 24 h, and then embedded in paraffin. The LSG was sectioned at 10-mm intervals, and the neuronal number of LSG was determined using Cresyl violet staining (Solarbio, China). The cardiac sympathetic post-ganglionic cell bodies were counted and analyzed. Additionally, the expression of TH (anti-TH antibody, Abcam, Cambridge, England) in the LSG tissue was examined using immunofluorescence. After staining the tissue, three fields per slice were quantified and averaged in a blinded manner under 40X magnification. Image J software was used for quantitative analysis. The LSG were used for immunohistochemical staining to assess the apoptosis. The LSG slices were stained for Bcl-2 (1:100; CST) and Bax (1:100; CST). Tissues from different groups were stained simultaneously.

### Western Blotting

Western blotting analysis was used to assess the expression of apoptosis-related protein of the LSG. The total protein was extracted from the LSG tissue. The LSG tissues were lysed in RIPA extraction buffer (Beyotime, China) and then centrifuged at 12,000 rpm for 10 min at 4 ºC. The total protein concentration was measured by BCA Protein Assay Kit (TIANGEN, China). The LSG proteins were separated by electrophoresis in 12% SDS-PAGE and then transferred onto polyvinylidene difluoride (PVDF) membranes. The membranes were probed with antibodies against β-catenin (1:1000; Abcam), Bcl-2 (1:1000; CST), Bax (1:1000; CST), and glyceraldehydes-3-phosphate dehydrogenase (GAPDH 1:3000; Affinity). Next, the membranes were followed by incubation with secondary antibodies, and finally were exposed with ECL reagent (Proteintech, China), and scanned by the Imaging System. The content of β-catenin, Bcl-2 and Bax were normalized to GAPDH. A minimum of three independent WB experiments were performed, and the Image-lab 4.0.1 software was used for analysis.

### Statistical Analysis

Data in this study were expressed as mean ± standard deviations (M ± SD). The data were evaluated for a normal distribution by the Shapiro–Wilk normality test. The one-way ANOVA test was used when the data fulfilled the normality test, while the non-parametric Mann–Whitney test was used when the data failed the normality test. All of the data were analyzed using SPSS software version 27 (SPSS Inc., Chicago, IL, USA). *P* < 0.05 were defined statistically significant.

## Results

### MI Model Evaluation and LDTN Stimulation

The site of ligation of LAD was shown as Fig. 1C. After LADO, the ST-segment was elevated significantly in ECG lead II compared with the baseline. The VAs episodes were easily observed in most animals during the ischemic preconditioning and after the LADO process, including ventricular premature beats (VPB, defined as one or two consecutive VPBs), ventricular tachycardia (VT, defined as three or more consecutive VPBs), and ventricular fibrillation (VF) (Fig. 1B). Taken together, the canine MI model established by ligation of the LAD was successful and feasible. Then, the LDTN was successfully separated and stimulated immediately after LADO (Fig. 1D and F). During the whole period of LDTN stimulation of 2 months, none died and none of these beagles presented the symptoms of weight loss or appetite loss. One beagle died from refractory VF during the process of LADO.

### Effect of LDTN Stimulation on HRV Analysis

**Fig. 2:**
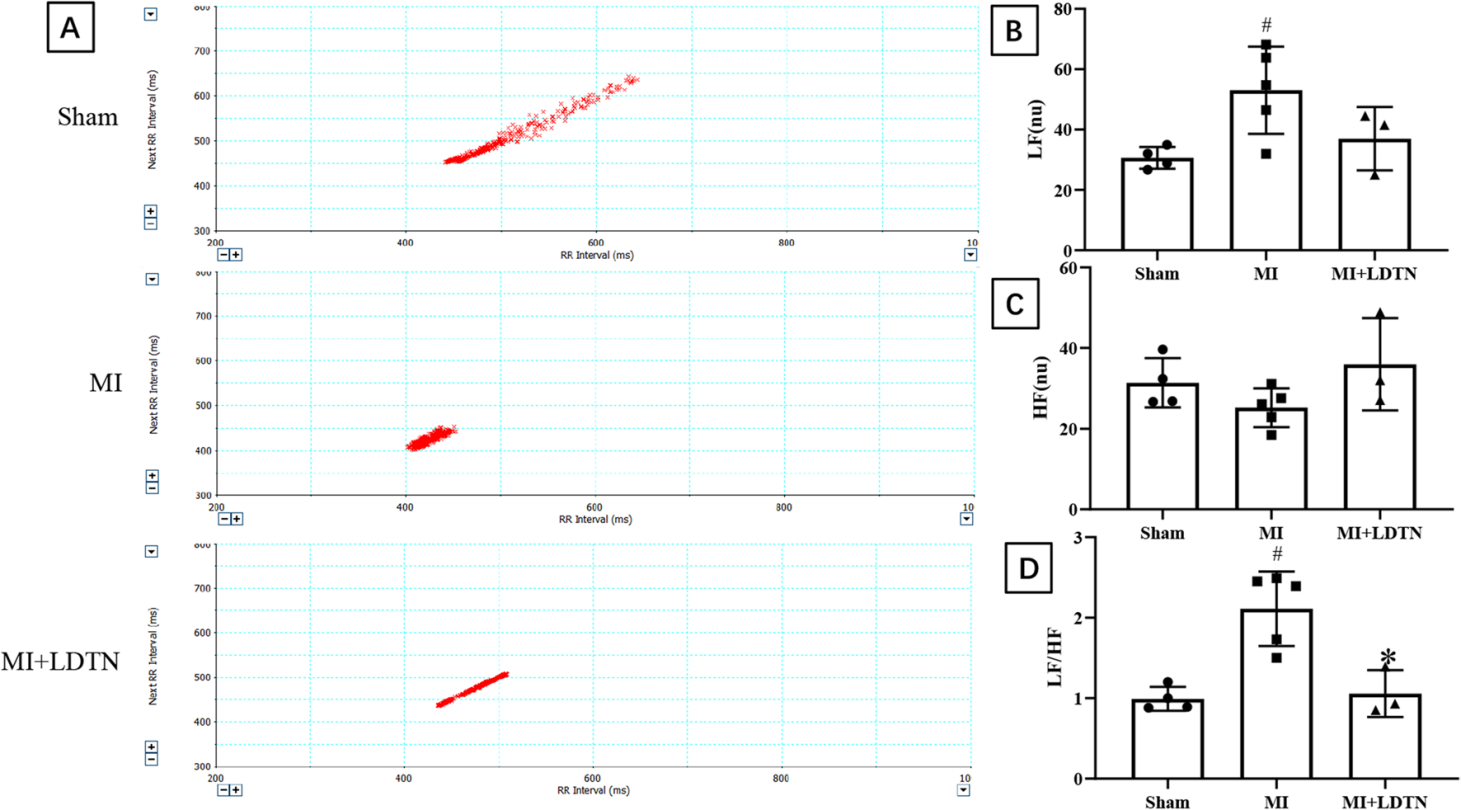
Effects of LDTN stimulation on HRV. **(A)** Representative images of the plot-Poincaré maps of each group. **(B)** and **(C)** and **(D)** The LF value, the HF value and the LF/HF ratio in each group, respectively. Data are shown as means ± SD. ^#^*P* < 0.05 versus the sham group; *P < 0.05 versus the MI group. HRV, heart rate variability; LF, low-frequency component; HF, high-frequency component

The representative images of Poincaré plot maps were shown in Fig. 2A. From the Poincaré plot maps, we intuitively found that the HRV of the MI + LDTN group was decreased compared with that of the MI group. Moreover, spectrum analysis (LF, HF, and LF/HF ratio) was performed to evaluate the LDTN stimulation on specific components of the autonomic nervous system. In addition, the spectrum analysis showed that the LF (nu) and LF/HF ratio were significantly increased in the MI group compared with the control group (LF 53.03 ± 14.46 versus 30.63 ± 3.61, *P* < 0.05; and LF/HF ratio 2.11 ± 0.46 versus 1.00 ± 0.15, *P* < 0.05, respectively) (Fig. 2B and D), while HF (nu) was slightly reduced (25.19 ± 4.77 versus 31.34 ± 6.13, *P* > 0.05) in the MI group (Fig. 2C); however, LDTN stimulation decreased the LF/HF ratio (LF/HF ratio 1.06 ± 0.29 versus 2.11 ± 0.46, *P* < 0.05), the LF (nu) value showed a decreasing trend (LF 36.99 ± 10.50 versus 53.03 ± 14.46, *P* > 0.05) and the HF (nu) showed an increasing trend (HF 35.95 ± 11.48 versus 31.34 ± 6.13, *P* > 0.05).

### Effect of LDTN Stimulation on Ventricular Electrophysiological Properties

**Fig. 3:**
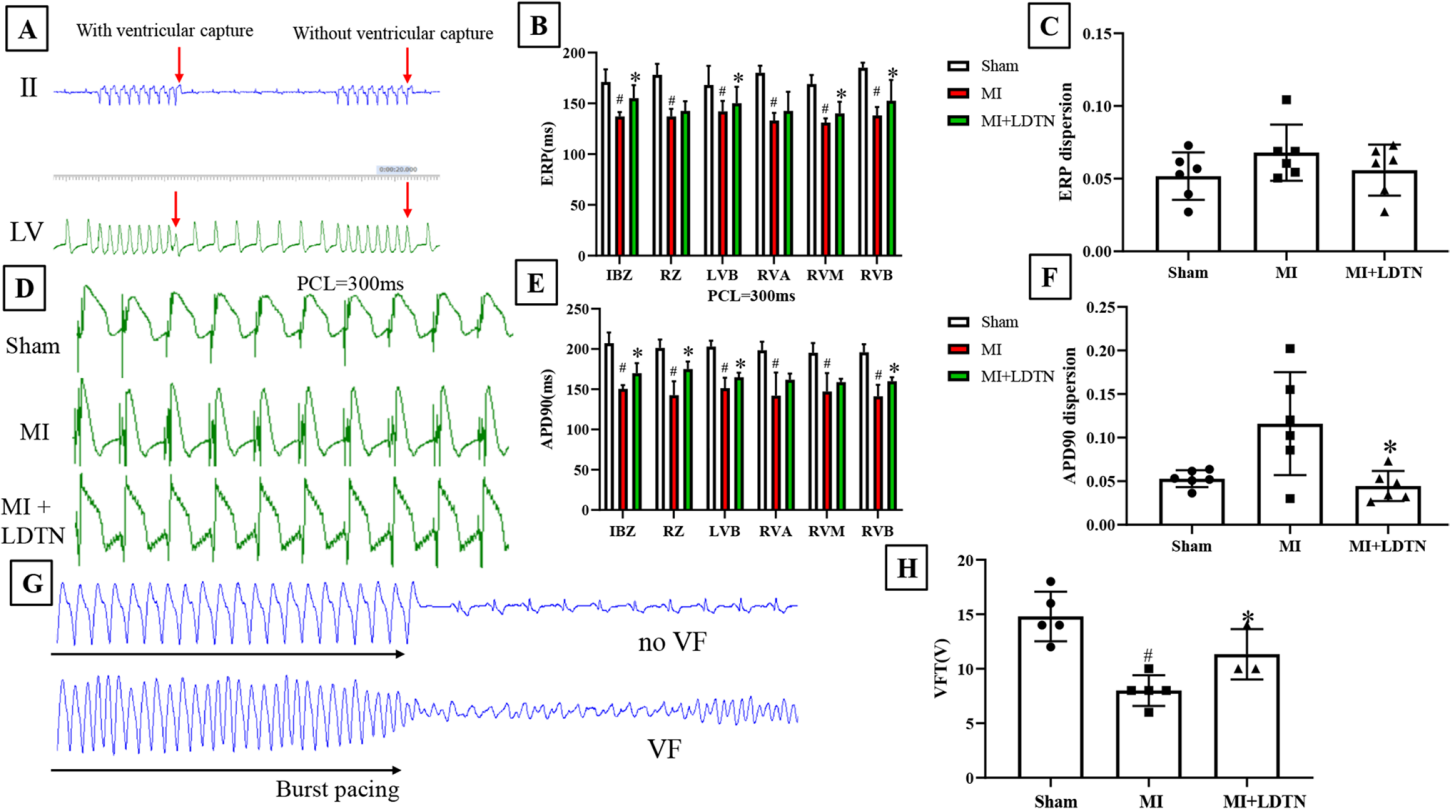
Effects of LDTN stimulation on ventricular parameters. **(A)** Representative images of ERP measurement; **(B)** The ERP values at six epicardial sites; **(C)** The disperse of ERP in each group; **(D)** Representative images of MAP at PCL of 300 ms in each group; **(E)** The APD90 values at six epicardial sites; **(F)** The disperse of APD90 in each group. **(G)** Representative images of burst pacing of the IBZ to induce VF. **(H)** Quantitative analysis of VFT in each group. Data are shown as means ± SD. ^#^*P* < 0.05 versus the sham group; *P < 0.05 versus the MI group. ERP, effective refractory period; MAP, monophasic action potential; APD90, action potential duration at 90% repolarization; VF, ventricular fibrillation; VFT, ventricular fibrillation threshold; IBZ, infarct border zone; RZ, remote zone; LVB, left ventricular base; RVA, right ventricular apex; RVM, the median right ventricular; RVB, left ventricular base

The representative images of ERP measurement and monophasic action potentials (MAP) of IBZ in each group were shown in Fig. 3A and D, respectively. Compared with the sham group, ERP and APD90 in the MI group have shown a trend of shortening (all *P* < *0.05*) (Fig. 3B and E), and the dispersion of ERP and APD90 showed an increasing trend (Fig. 3C and F); however, LDTN stimulation reversed these trends (Fig. 3B, C and E, F). Representative images of burst pacing of the IBZ to induce VF is shown in Fig. 3G. In comparison with the sham group, VFT in the MI group was significantly reduced (8.00 ± 1.41 V versus 14.80 ± 2.28 V, *P* < 0.05); however, LDTN stimulation reversed this trend (11.33 ± 2.31 V versus 8.00 ± 1.41 V, *P* < 0.05) (Fig. 3H).

### Effect of LDTN Stimulation on Neural Activity and Number of Neurons of LSG

**Fig. 4:**
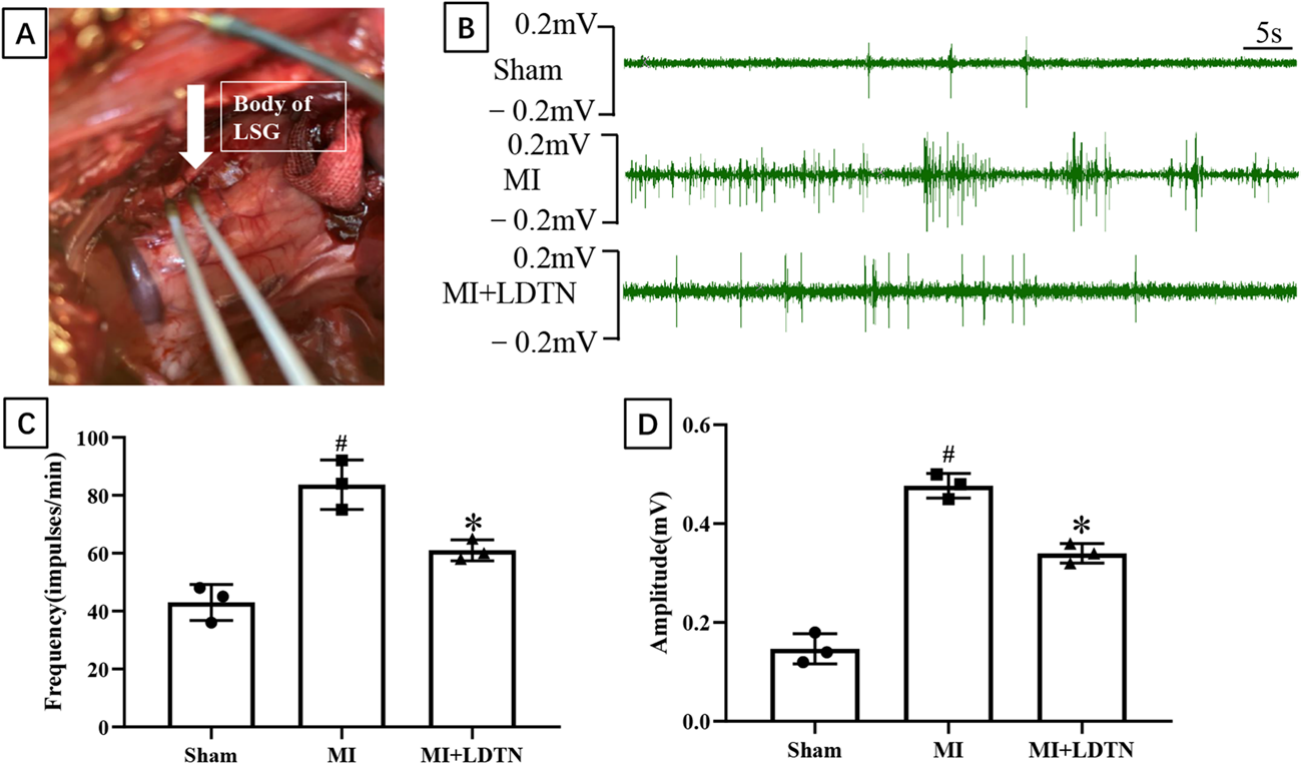
Effects of LDTN stimulation on the LSG function and neural activity. **(A)** Representative images of anatomical structures of the LSG. The white arrow indicates the body of LSG for detecting the neural activity of LSG. **(B)** Representative images of the neural activity of LSG in each group. **(C)** and **(D)** Quantitative analysis of the discharge frequency and amplitude of the LSG, respectively. Data are shown as means ± SD. ^#^*P* < 0.05 versus the sham group; *P < 0.05 versus the MI group

**Fig. 5:**
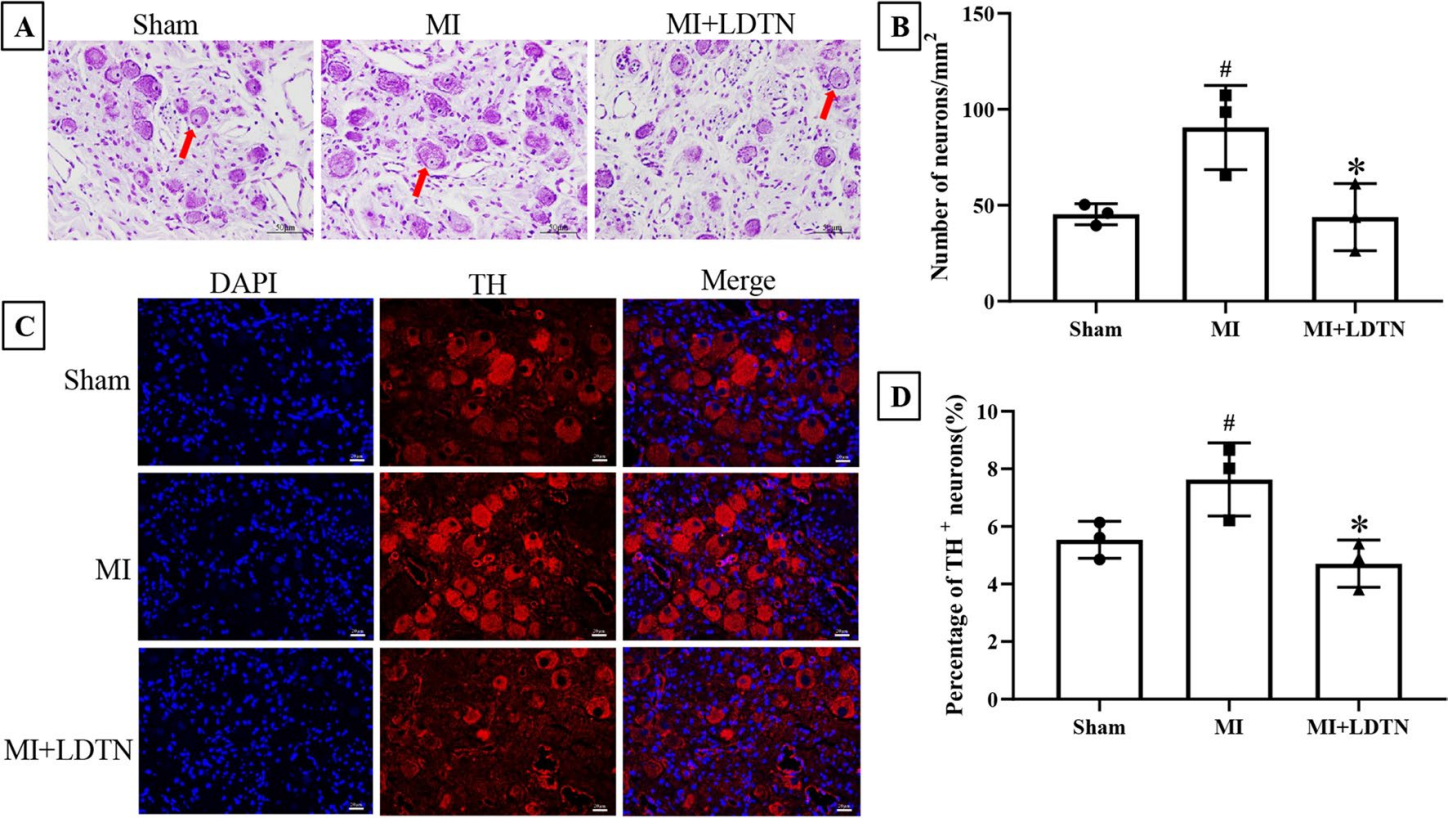
Effects of LDTN stimulation on the number of neurons in the LSG tissue. **(A)** Representative images of cresyl violet-stained neurons of the LSG. Red arrows represent the bodies of LSG neurons. Scale bars 50 μm. **(B)** Counts of LSG neurons. #*P* < 0.05 versus the sham group; *P < *0.05* versus the MI group. **(C)** Representative images of IF staining of TH (red). The nuclei were stained with DAPI (blue). Scale bars 20 μm. **(D)** The percentage of TH-positive neurons; #*P* < 0.05 versus the sham group; *P < 0.05 versus the MI group. IF, immunofluorescence; TH, tyrosine hydroxylase

The representative image of the anatomical location of the LSG was as shown in Fig. 4A. The representative images of LSG neural activity in each group were shown in Fig. 4B. Quantitative analysis showed a significant increase in both discharge frequency (83.67 ± 8.50 versus 43.00 ± 6.24 impulses/min, *P* < 0.05) and discharge amplitude (0.48 ± 0.02 versus 0.15 ± 0.03 mV, *P* < 0.05) of the LSG nerve in the MI group compared with the sham group; however, LDTN stimulation decreased discharge frequency (61.00 ± 3.60 versus 83.67 ± 8.50 impulses/min, *P* < 0.05) and discharge amplitude (0.34 ± 0.02 versus 0.48 ± 0.02 mV, *P* < 0.05) compared with the MI group (Fig. 4C and D). Additionally, histological staining and immunofluorescence was used to assess the effect of LDTN stimulation on number of neurons of the LSG. The representative images of cresyl violet and TH immunofluorescence staining of LSG were shown in Fig. 5A and C. The number of LSG neurons in the MI group was increased compared with that of the sham group (90.52 ± 21.93/mm^2^ versus 45.36 ± 5.51/mm^2^, *P* < 0.05); however, the number of LSG neurons in the MI + LDTN group was decreased compared with that of the MI group (43.80 ± 17.52/mm^2^ versus 90.52 ± 21.93/mm^2^, *P* < 0.05) (Fig. 5B). Compared with the sham group, the percentage of TH-positive neurons of LSG was increased (7.41 ± 1.64% versus 5.54 ± 0.64%, *P* < 0.05); however, LDTN stimulation decreased the percentage of TH-positive neurons (4.71 ± 0.81% versus 7.41 ± 1.64%, *P* < 0.05) (Fig. 5D).

### Effect of LDTN Stimulation on LSG Neuronal Apoptosis

**Fig. 6:**
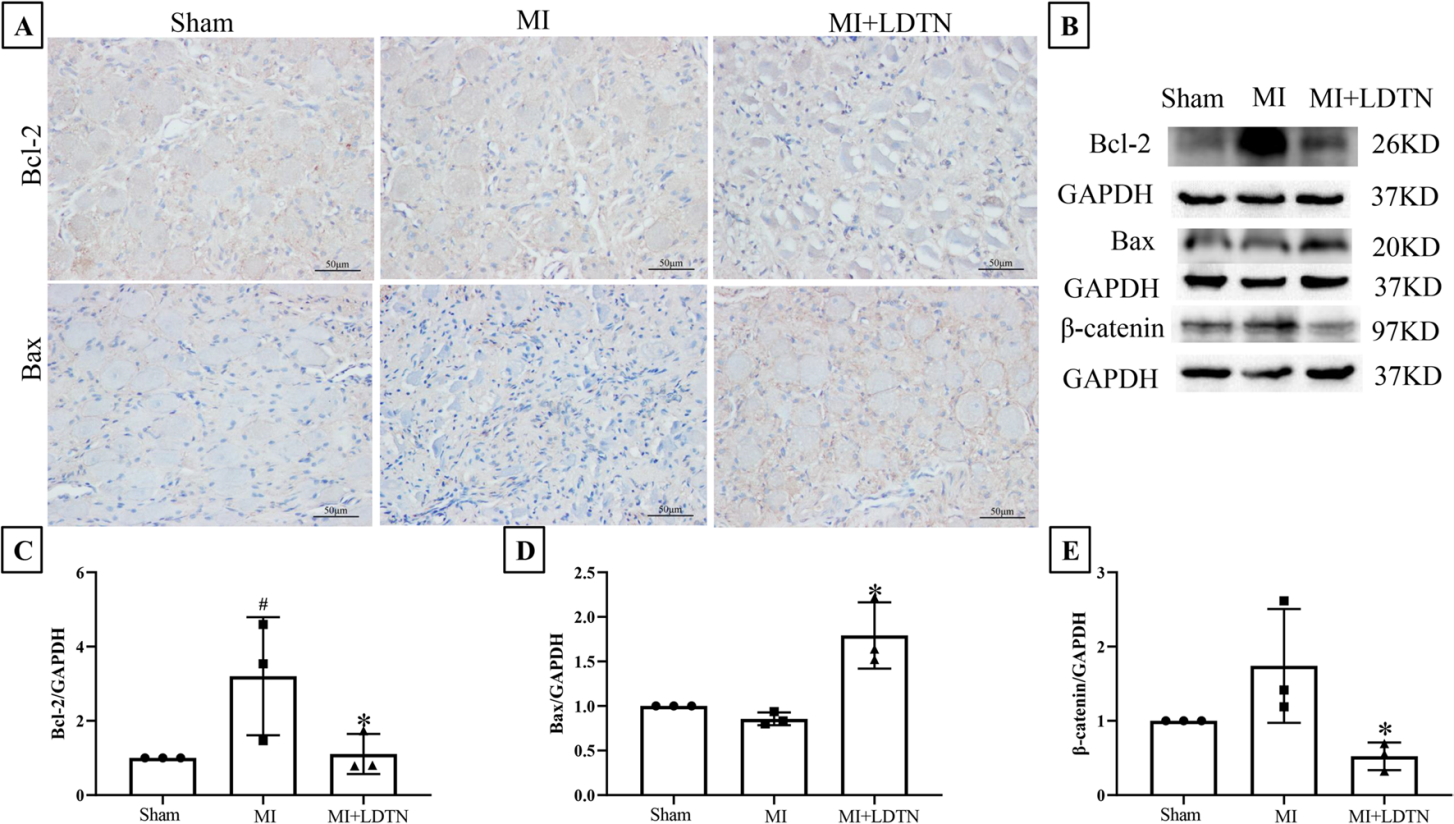
Effects of LDTN stimulation on LSG neuronal apoptosis. **(A)** Representative images of immunohistochemical staining of apoptosis-related proteins (Bcl-2 and Bax) of the LSG tissue. **(B)** WB analysis of apoptosis-related proteins of the LSG tissue. **(C)** and **(D)** and **(E)** Quantitative analysis of Bcl-2, Bax, and β-catenin, respectively. Data are shown as means ± SD. ^#^*P* < 0.05 versus the sham; *P < 0.05 versus MI. Scale bar 50 μm. WB, western blotting

To confirm whether LDTN stimulation leads to damage to the LSG, we used IHC staining to detect the expression of apoptosis-related proteins. The representative images of IHC of Bcl-2 and Bax were shown in Fig. 6A. We found that the pro-apoptotic protein Bax was increased and the anti-apoptotic protein Bcl-2 was decreased in the MI + LDTN group compared with these of the MI group. Additionally, WB assay was performed to further determine the expression levels of apoptosis-related proteins (Bcl-2 and Bax) (Fig. 6B). Quantity analysis showed that the pro-apoptotic protein Bax was increased and the anti-apoptotic protein Bcl-2 of the LDTN group was decreased compared with these of the MI group (all *P* < 0.05) (Fig. 6C and D). Furthermore, the β-catenin was decreased in the MI + LDTN group compared with that of the MI group (*P* < 0.05) (Fig. 6E).

## Discussion

In this study, we initially tried to evaluate the effect of long-term LDTN stimulation on ventricular electrophysiological stability and further explored its possible mechanisms in a canine model of chronic MI. We found that the increased sympathetic components of HRV and LSG neuronal discharge activity were suppressed by LDTN stimulation. In addition, LDTN stimulation resulted in a considerable reduction in VAs susceptibility by prolonging ventricular ERP and APD90, decreasing the dispersion of ERP and APD90, and increasing VFT. Notably, the increased neural apoptosis of LSG was observed in the MI + LDTN group compared with that in the other groups. Taken together, our study provides direct evidence for a potential role of LDTN stimulation in improving ventricular electrical remodeling in a canine model of chronic MI through neural apoptosis of LSG.

It is widely recognized that the autonomic nervous system plays a key role in regulation of cardiac electrophysiology. Briefly, sympathetic activation results in the instability of cardiac electrophysiology; conversely, sympathetic inhibition or vagus nerve activation exhibits an opposite effect [13, 14]. The Poincaré plot of R–R intervals is a geometrical method for visually and quantitatively describing the heart rate variability [15]. The LF value indicates the sympathetic tone, and the HF value indicates the parasympathetic tone, while the LF/HF ratio indicates the relative balance between sympathetic and parasympathetic nerves [16]. In this present study, a significant increase in the LF (nu) value and LF/HF ratio, and a reduction in HF (nu) value were observed in the MI group compared with that in the sham group. However, LDTN stimulation led to a significant reduction in the LF (nu) value and LF/HF ratio and an increase in the HF (nu) value. These results indicated that LDTN stimulation can improve the imbalance of autonomic nervous system function.

It is well known that the function and activity of LSG play a key role in the regulation of ventricular electrophysiology; therefore, we evaluated the effect of LDTN stimulation on the function and activity of LSG. In this study, the LSG discharge activity was increased in the MI group compared with that in the sham group; however, the LDTN stimulation decreased the LSG discharge activity. The results indicated that LDTN stimulation could inhibit the LSG activity. Furthermore, cresyl violet staining results showed that LDTN stimulation decreased the number of neurons, while IF staining results showed that LDTN stimulation decreased the percentage of TH positive neurons. Taken together, our results indicated that LDTN stimulation could inhibit the LSG function and activity, which may be a novel method for heart sympathetic denervation; however, this still needs further investigation and verification.

Previous studies indicated that sympathetic stimulation induces a decrease in ventricular ERP and APD, along with an increase in dispersion of repolarization [17, 18]. Thus, we evaluated the effects of LDTN stimulation on ventricular electrophysiological properties in this study. We found that the ERP and APD90 were decreased in the MI group, and the dispersions were increased or showed an increasing trend; however, the LDTN stimulation prolonged the ERP and APD90 and decreased their dispersions. The results indicated that ventricular electrophysiological properties were improved by LDTN stimulation. The possible reason may be that the protective effect of LDTN stimulation on ventricular electrical remodeling can be partly resulted from the regulation of sympathetic remodeling. However, further investigation is required to elucidate the exact molecular mechanism underlying the improvement in ventricular electrical remodeling following LDTN stimulation.

As mentioned above, LDTN stimulation decreased the activity of LSG and the percentage of TH positive cells. Therefore, we aimed to identify whether the above results were caused by the apoptosis of LSG neurons. Previous studies have shown that vagus nerve stimulation can cause neuronal damage in the stellate ganglion. It is mainly due to the fact that vagus nerve fibers contain some sympathetic nerve fibers originated from the stellate ganglion. However, stimulating the vagus nerve increases the excitability of the stellate ganglion [8, 19]. The rapid and sustained stimulation can lead to the excitotoxicity of neurons and therefore result in damage and apoptosis to neurons [20]. The LDTN is connected to the LSG and contains the postganglionic sympathetic nerve components originating from the LSG. A recent study has shown that long-term stimulation of LDTN leads to damage of the LSG neurons and therefore attenuates its activity [7]. Consistent with previous findings, our results showed that LDTN stimulation also inhibited the LSG discharge activity; however, the underlying molecular mechanism has not been elucidated. Therefore, we subsequently identify whether LDTN stimulation inhibiting LSG activity was related to neuronal apoptosis and explored the exact molecular mechanisms.

The Bcl-2 protein family is well recognized to regulate mitochondrial integrity and apoptosis at each physiological or functional level, including pro-apoptotic and anti-apoptotic members [21]. A recent study showed that Shuxuening injection can reduce the apoptosis of hippocampal neurons induced by cerebral ischemia–reperfusion injury in rats by inhibiting the activation of Bax/Bcl-2 (22). Another study has shown that leptin protects against hyperglycemia-induced neural damage by inhibiting Bax/Bcl-2 ratio [23]. In our study, the IHC result showed that LDTN stimulation increased the expression of pro-apoptotic Bax and reduced the expression of anti-apoptotic Bcl-2. Furthermore, the WB assay results were consistent with the IHC results. These results collectively indicated that LDTN stimulation increased the pro-apoptotic Bax, and decreased the anti-apoptotic Bcl-2, thereby resulting in the LSG apoptosis. Additionally, the β-catenin was increased in the MI group compared with that in the sham group. Conversely, the MI + LDTN group exhibited a greater reduction in β-catenin levels compared with the MI group. These results indicated that LDTN stimulation could inhibit the activated Wnt/β-catenin signaling pathway, thereby resulting in the neuronal apoptosis of LSG through up-regulation of pro-apoptotic Bax, and down-regulation of anti-apoptotic Bcl-2.

Together, we initially aimed to investigate the effect of LDTN stimulation on ventricular arrhythmias after MI, and finally found that the LDTN stimulation could improve ventricular electrical remodeling and exhibit some cardioprotective effect on the heart by inhibiting the discharge of LSG. Additionally, the LDTN is a subcutaneous nerve, which is simple to isolate with less damage and has some advantages when compared with traditional SG block or resection if LDTN stimulation is further defined clinically effective. However, the present study has several potential limitations. First, the sample size of experimental animals is relatively small. Although beagle dogs with similar age and weight were selected for the experiment, there remain some individual differences, which need to be further studied by expanding the sample size. Second, some in vivo experiments are carried out under anesthesia in dogs, which may have certain effects on the heart rate and blood pressure of experimental animals. However, all experimental animals have similar anesthesia methods and degrees, so the effects of anesthesia can be almost offset between groups. Third, there are some differences between beagles and human structure and the tolerance, which may limit the interpretation of experimental results. In addition, the optimal parameters, duration, and timing of LDTN stimulation were not explored. Of course, these are also the direction of our future research. We will expand the sample size of the study, deepen the mechanism research, and conduct further research on different parameters, different stimulation duration and optimal stimulation timing.

## Conclusions

LDTN stimulation attenuates sympathetic remodeling by suppressing the activated Wnt/β-catenin signaling pathway, thereby triggering LSG neuronal apoptosis, and improving ventricular electrical remodeling. LDTN stimulation may be a novel approach to prevent ventricular arrhythmias and SCD following MI.

## Data Availability

The data that support the findings of this study are available from the corresponding author upon reasonable request.

## References

[1] Hayashi M, Shimizu W, Albert CM. The spectrum of epidemiology underlying sudden cardiac death. Circ Res. 2015;116(12):1887–906. 10.1161/CIRCRESAHA.116.304521 26044246 10.1161/CIRCRESAHA.116.304521 PMC4929621

[2] Zhou S, Cao JM, Tebb ZD, et al. Modulation of QT interval by cardiac sympathetic nerve sprouting and the mechanisms of ventricular arrhythmia in a canine model of sudden cardiac death. J Cardiovasc Electrophysiol. 2001;12(9):1068–73. 10.1046/j.1540-8167.2001.01068.x 11573698 10.1046/j.1540-8167.2001.01068.x

[3] Chen PS, Chen LS, Cao JM, et al. Sympathetic nerve sprouting, electrical remodeling and the mechanisms of sudden cardiac death. Cardiovasc Res. 2001;50(2):409–16. 10.1016/s0008-6363(00)00308-4 11334845 10.1016/s0008-6363(00)00308-4

[4] Herring N, Kalla M, Paterson DJ. The autonomic nervous system and cardiac arrhythmias: current concepts and emerging therapies. Nat Rev Cardiol. 2019;16(12):707–26. 10.1038/s41569-019-0221-2 31197232 10.1038/s41569-019-0221-2

[5] Xiong L, Liu Y, Zhou M, et al. Targeted ablation of cardiac sympathetic neurons improves ventricular electrical remodelling in a canine model of chronic myocardial infarction. Europace. 2018;20(12):2036–44. 10.1093/europace/euy090 29860489 10.1093/europace/euy090

[6] Xiong L, Liu Y, Zhou M, et al. Targeted ablation of cardiac sympathetic neurons attenuates adverse postinfarction remodelling and left ventricular dysfunction. Exp Physiol. 2018;103(9):1221–9. 10.1113/EP086928 29928790 10.1113/EP086928

[7] Zhao Y, Yuan Y, Tsai WC, et al. Antiarrhythmic effects of stimulating the left dorsal branch of the thoracic nerve in a canine model of paroxysmal atrial tachyarrhythmias. Heart Rhythm. 2018;15(8):1242–51. 10.1016/j.hrthm.2018.04.009 29654853 10.1016/j.hrthm.2018.04.009 PMC6067960

[8] Chinda K, Tsai WC, Chan YH, et al. Intermittent left cervical vagal nerve stimulation damages the stellate ganglia and reduces the ventricular rate during sustained atrial fibrillation in ambulatory dogs. Heart Rhythm. 2016;13(3):771–80. 10.1016/j.hrthm.2015.11.031 26607063 10.1016/j.hrthm.2015.11.031 PMC4762711

[9] Abou Ziki MD, Mani A. Wnt signaling, a novel pathway regulating blood pressure? State of the art review. Atherosclerosis. 2017;262:171–8. 10.1016/j.atherosclerosis.2017.05.001 28522145 10.1016/j.atherosclerosis.2017.05.001 PMC5508596

[10] Gao K, Wang YS, Yuan YJ, et al. Neuroprotective effect of rapamycin on spinal cord injury via activation of the Wnt/β-catenin signaling pathway. Neural Regen Res. 2015;10(6):951–7. 10.4103/1673-5374.158360 26199613 10.4103/1673-5374.158360 PMC4498358

[11] Zhang L, Cheng H, Yue Y, et al. H19 knockdown suppresses proliferation and induces apoptosis by regulating miR-148b/WNT/β-catenin in ox-LDL-stimulated vascular smooth muscle cells. J Biomed Sci. 2018;25(1):11. 10.1186/s12929-018-0418-4 29415742 10.1186/s12929-018-0418-4 PMC5804091

[12] Garan H, Fallon JT, Ruskin JN. Sustained ventricular tachycardia in recent canine myocardial infarction. Circulation. 1980;62(5):980–7. 10.1161/01.cir.62.5.980 7418182 10.1161/01.cir.62.5.980

[13] Hou Y, Zhou Q, Po SS. Neuromodulation for cardiac arrhythmia. Heart Rhythm. 2016;13(2):584–92. 10.1016/j.hrthm.2015.10.001 26440550 10.1016/j.hrthm.2015.10.001

[14] Chen PS, Choi EK, Zhou S, et al. Cardiac neural remodeling and its role in arrhythmogenesis. Heart Rhythm. 2010;7(10):1512–3. 10.1016/j.hrthm.2010.05.020 20478404 10.1016/j.hrthm.2010.05.020 PMC2946520

[15] Brennan M, Palaniswami M, Kamen P. Poincaré plot interpretation using a physiological model of HRV based on a network of oscillators. Am J Physiol Heart Circ Physiol. 2002;283(5):H1873-1886. 10.1152/ajpheart.00405.2000 12384465 10.1152/ajpheart.00405.2000

[16] Gorman JM, Sloan RP. Heart rate variability in depressive and anxiety disorders. Am Heart J. 2000;140(4 Suppl):77–83. 10.1067/mhj.2000.109981 11011352 10.1067/mhj.2000.109981

[17] Ng GA, Mantravadi R, Walker WH, et al. Sympathetic nerve stimulation produces spatial heterogeneities of action potential restitution. Heart Rhythm. 2009;6(5):696–706. 10.1016/j.hrthm.2009.01.035 19389655 10.1016/j.hrthm.2009.01.035

[18] Ng GA, Brack KE, Patel VH, et al. Autonomic modulation of electrical restitution, alternans and ventricular fibrillation initiation in the isolated heart. Cardiovasc Res. 2007;73(4):750–60. 10.1016/j.cardiores.2006.12.001 17217937 10.1016/j.cardiores.2006.12.001

[19] Rhee KS, Hsueh CH, Hellyer JA, et al. Cervical vagal nerve stimulation activates the stellate ganglion in ambulatory dogs. Korean Circ J. 2015;45(2):149–57. 10.4070/kcj.2015.45.2.149 25810737 10.4070/kcj.2015.45.2.149 PMC4372981

[20] Nicholls DG, Ward MW. Mitochondrial membrane potential and neuronal glutamate excitotoxicity: mortality and millivolts. Trends Neurosci. 2000;23(4):166–74. 10.1016/s0166-2236(99)01534-9 10717676 10.1016/s0166-2236(99)01534-9

[21] Tsujimoto Y. Cell death regulation by the Bcl-2 protein family in the mitochondria. J Cell Physiol. 2003;195(2):158–67. 10.1002/jcp.10254. 12652643 10.1002/jcp.10254

[22] Li Z, Xiao G, Wang H, et al. A preparation of Ginkgo biloba L. leaves extract inhibits the apoptosis of hippocampal neurons in post-stroke mice via regulating the expression of Bax/Bcl-2 and Caspase-3. J Ethnopharmacol. 2021;280:114481. 10.1016/j.jep.2021.114481 10.1016/j.jep.2021.114481 34343651

[23] Kaeidi A, Hajializadeh Z, Jahandari F, et al. Leptin attenuates oxidative stress and neuronal apoptosis in hyperglycemic condition. Fundam Clin Pharmacol. 2019;33(1):75–83. 10.1111/fcp.12411 30203422 10.1111/fcp.12411

